# A Case of Acute Left Main Coronary Obstruction Following Transcatheter Aortic Valve Implantation

**DOI:** 10.7759/cureus.1951

**Published:** 2017-12-14

**Authors:** Iva N Dimitrova, Diana Trendafilova, Peyo Simeonov

**Affiliations:** 1 Department of Cardiology, University Hospital St. Ekaterina, Medical University of Sofia, Bulgaria

**Keywords:** coronary obstruction, transcatheter aortic valve implantation

## Abstract

Transcatheter aortic valve implantation (TAVI) is a highly effective procedure in selected patients with severe degenerative aortic valve stenosis at high risk for conventional surgery. Coronary occlusion is a periprocedural life-threatening complication that despite its low frequency (˂1%) is poorly predictable and requires immediate diagnosis and treatment. Herein, we report a coronary obstruction after transcatheter implantation of valve prosthesis, followed by coronary intervention with successful recanalization.

## Introduction

Transcatheter aortic valve implantation (TAVI) is an excellent alternative for patients with severe aortic stenosis considered to be at high risk for open heart surgery because of advanced age and numerous accompanying diseases [1]. Nowadays, TAVI is considered as the only alternative to cardiac surgery with excellent long term results [2]. However, TAVI procedures have potential severe complications, and one of them is coronary ostium obstruction, which could be a fatal, life-threatening complication that needs urgent diagnosis and treatment [3].

We report a rare case of a transfemoral aortic valve implantation complicated by acute left main coronary artery (LMCA) occlusion and hemodynamic collapse, which was successfully treated by balloon angioplasty and stent implantation.

## Case presentation

An 80-year-old man presented to our cardiology department with symptoms of advanced heart failure (New York Heart Association IV Functional Class), cardiac asthma at rest, bilateral pleural effusions, peripheral oedema, and symptoms of syncope. The accompanying diseases were anaemia, stage I of chronic kidney disease, and chronic obstructive pulmonary disease. Electrocardiography (ECG) showed atrial fibrillation with significant pauses. The transthoracic echocardiogram revealed severe aortic valve stenosis (max transvalvular gradient 83 mmHg, mean gradient 64 mmHg, aortic valve area 0.62 cm^2^), mild mitral regurgitation, severe tricuspidal regurgitation, elevated pulmonary artery systolic pressure, and left ventricular ejection fraction (LVEF) of 50%. A coronary angiogram displayed significant right coronary artery and obtuse marginal artery 1 stenosis.

**Figure 1:**
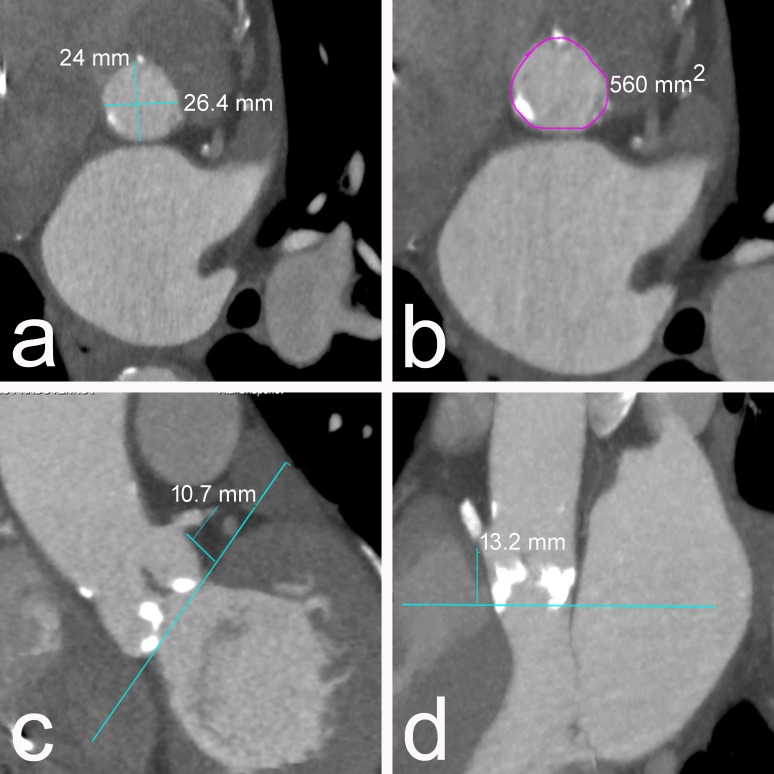
Preprocedural CT evaluation. CT - computed tomography

We calculated a logistic EuroSCORE of 24.9%, a Society of Thoracic Surgeons risk score (STS) of 18.6%, a EuroSCORE II of 7.8% and considered that the patient is at high risk for conventional surgery, and after a heart team discussion, he was referred to TAVI. A transesophageal echocardiogram showed a tricuspid aortic valve and bulky calculus on and between the leaflets. The aortic annular size was 24-25 mm and the sinus of Valsalva was 33 mm. Multislice computed tomography (CT) showed an aortic annulus size of 26.4/24 mm (Figure 1a), sinus of Valsalva size of 33 mm, annulus area of 560 mm^2^ (Figure 1b), bulky, heavily calcified leaflets, high of LMCA 10.7 mm (Figure 1c), and right coronary artey (RCA) of 13.2 mm (Figure 1d).

**Figure 2:**
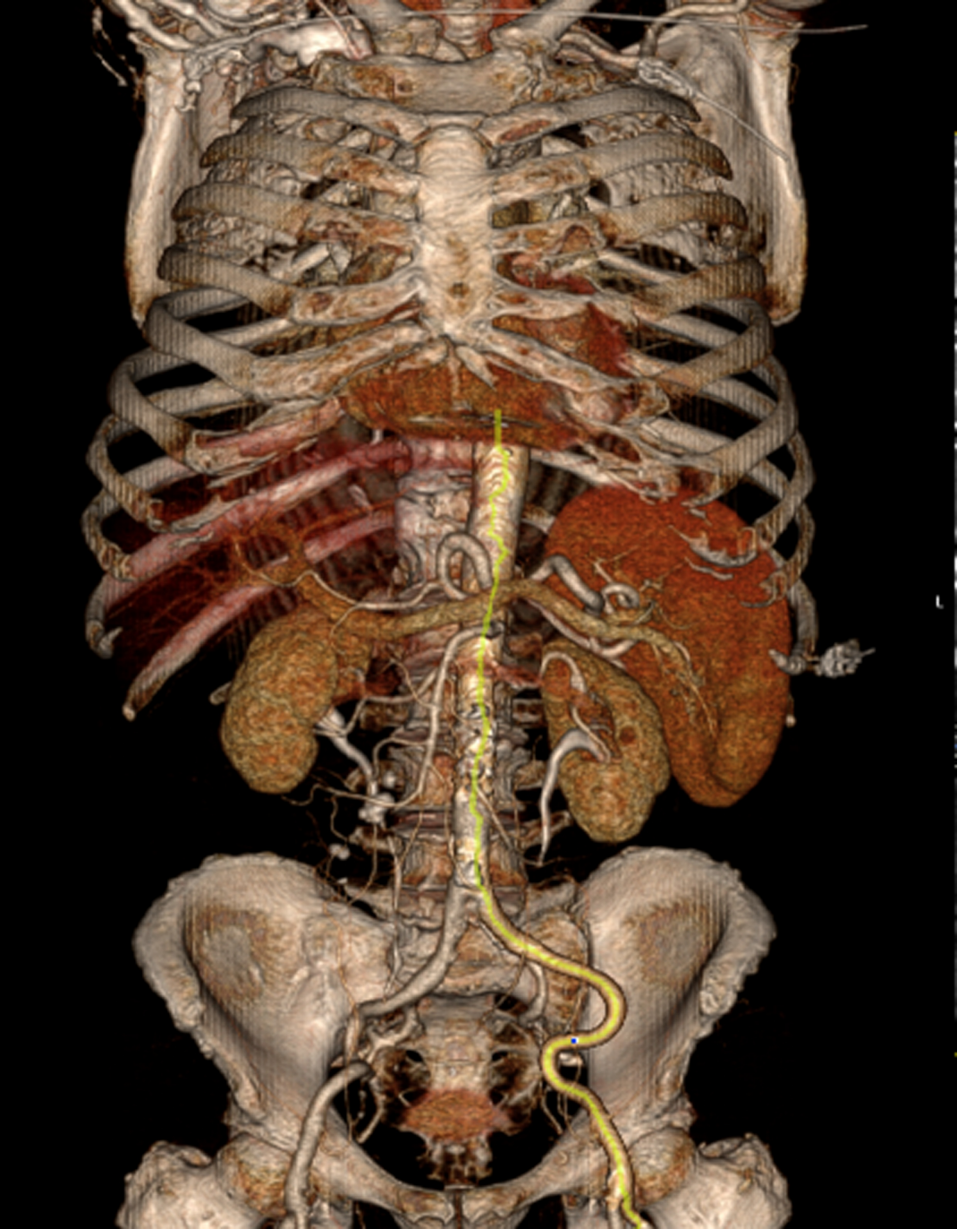
Multislice CT angiography presents iliofemoral arterial segment - suitable for transfemoral approach. CT - computed tomography

CT imaging of lower extremity arteries (ilio-femoral arterial segment) revealed the following: right side - external iliac artery without significant stenoses, common femoral artery - 9.9/7.9 mm - without stenoses; left side - tortuous external iliac artery without significant stenoses, common femoral artery - 8/10 mm - without stenoses (Figure 2).

Based on the CT data, we chose the transfemoral approach.

**Figure 3:**
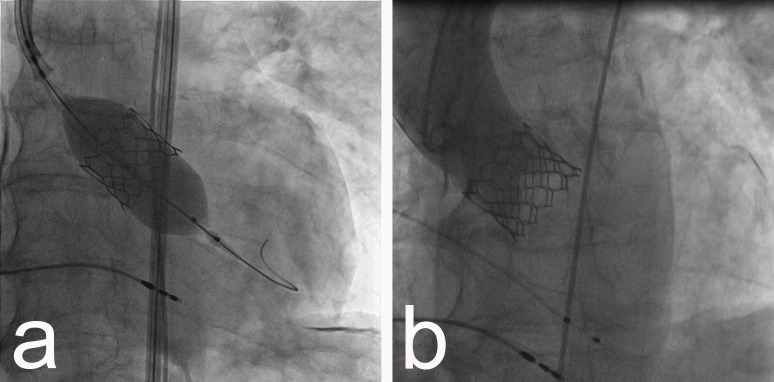
a) Implantation of 29 mm Edwards Sapien 3 valve; b) aortography showing prompt TAVI position with no paravalvular regurgitation. TAVI - transcatheter aortic valve implantation

After permanent pacemaker implantation and complete coronary interventional revascularization, a TAVI procedure was performed: under general anesthesia, a 16F sheath was inserted in the right femoral artery and after passing with a guidewire through the stenotic aortic valve, an inflation with a balloon 23/40 mm was done. Then a 29 mm Edwards Sapien 3 valve (Edwards Lifesciences, CA, USA) was implanted with good result (Figures 3a-3b).

**Figure 4:**
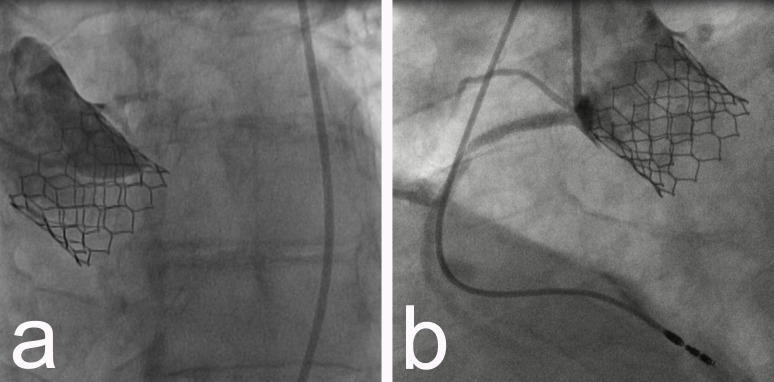
a) Angiography showing ostial LMCA occlusion; b) patient RCA. LMCA - left main coronary artery, RCA - right coronary artery

The patient was transferred to the intensive care unit, where one hour later he presented worsening hypotension (RR-50/30 mmHg), followed by ventricular fibrillation. An ECG performed after shock defibrillation showed periods of new left bundle branch block and periods of pacemaker rhythm. An echocardiogram revealed severe impaired LVEF with septo-apical hypokinesis. An urgent angiogram was performed and an LMCA ostial calcific obstruction was apparent in the angiogram: thrombolysis in myocardial infarction (TIMI) 0 flow in left anterior descending (LAD) and circumflex artery (Cx), and patient right coronary artery (RCA) (Figures 4a-4b).

**Figure 5:**
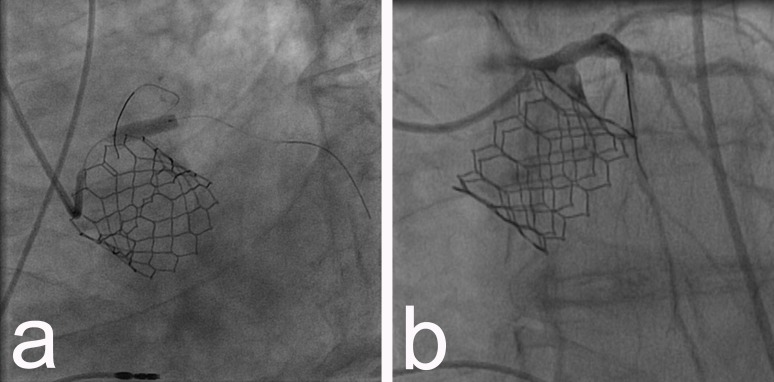
a) Placement of a stent in the LMCA; b) subsequent patency. LMCA - left main coronary artery

A 7F 4 extra backup (XB) guiding catheter was promptly positioned in the LMCA. Then, a floppy guidewire was advanced across the occlusion, and a 2.5/15 mm balloon was inflated. The procedure was completed by implanting a 4.5 /13 mm drug eluting stent (DES) (Figure 5a), which was finally postdilatated with a 5.0/15 mm balloon. This resulted in the restoration of TIMI grade 3 flow in LMCA, LAD, and Cx (Figure 5b).

**Figure 6:**
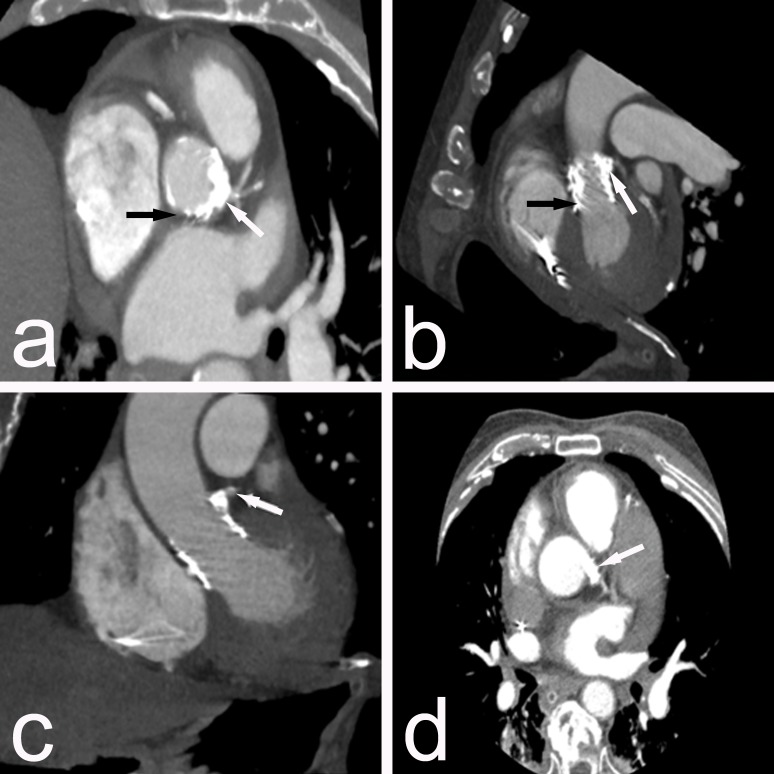
Control multidetector CT. a, b) showing Edwards Sapien 3 valve (black arrows) and protrusion of calcified valve leaflets in the left coronary sinus (white arrows); c, d) patient stent in LMCA (white arrows). CT - computed tomography, LMCA - left main coronary artery

An intra-aortic balloon pump (IABP) in assist ratio 1:1 was implanted. After recanalization, stable hemodynamic conditions were rapidly obtained. IABP was removed on the second day and catecholamines on the fourth day. A control multidetector CT was performed, which revealed a protrusion of calcified valve leaflets in the left coronary sinus, patient stent in LMCA, and prompt TAVI position (Figures 6a-6d). 

The patient was discharged on day 8, without signs of heart failure, without angina, and with medical therapy - vitamin K antagonist, aspirin, clopidogrel, ACE-inhibitor, statin, beta blocker, diuretic, and proton pump inhibitor. The echocardiogram at discharge showed mild apical hypokinesia with LVEF 50%, mild mitral regurgitation, max transvalvular gradient of implanted aortic valve 17 mm/Hg, mean 6 mm/Hg, no aortic regurgitation, moderate tricuspidal regurgitation, and decrease of pulmonary systolic pressure in comparison with the state before the procedure. The clinical course after this procedure was uneventful. Eight months later the patient was stable without any complaints.

## Discussion

TAVI is reported to be a highly effective procedure for high risk patients for aortic valve replacement [4]. Although TAVI is reported as less invasive than open cardiac surgery, different severe complications can occur. Some of them, because of their relatively high frequency (conduction disturbances, vascular complications, cerebrovascular events, paravalvular regurgitation, etc.), are largely described. Coronary occlusion and aortic rupture are two rare but life-threatening complications, and because of their poor prognosis, it is important that the definition of risk factors and prevention are clarified [1-4]. Coronary occlusion was described for the first time in 2006 by Webb, et al. [5]. In a large multicenter registry, Ribeiro, et al. [6] reported frequency of coronary occlusion in about 0.6% of patients. The 30-day mortality rate after coronary artery occlusion remained very high–about 40.9%. This complication is more frequent in female patients, affects most commonly the left coronary artery, and the use of a balloon-expandable valve (SAPIEN, Edwards Lifesciences, Irvine, CA, US), as well as the valve-in-valve procedures in degenerated surgical bioprostheses are associated with higher incidence [2, 6-7].

Different patient-related factors for coronary obstruction during TAVI have been reported in the current literature. Crimi, et al. [1] summarized them as: 1) narrow aortic annulus; 2) shallow sinuses of Valsalva; 3) low coronary ostia; 4) large calculus deposits of the aortic valve; and 5) previous plaque of the left main artery. These authors also depicted operator-related factors: high positioning or too large a valve. Two mechanisms of coronary obstruction are described: the first and more common one is due to the displacement of the calcification from the native valve, which obstructs the coronary ostium; the second cause is more hypothetical–obstruction due to a portion of the TAVI frame placed over the coronary ostium [6].

## Conclusions

TAVI is a highly effective procedure in selected patients at high surgical risk. However, the incidence of severe complications should not be overlooked. Coronary occlusion during or immediately after TAVI is a life-threatening complication that, despite the low incidence, is poorly predictable and requires immediate diagnosis and treatment. Therefore, careful patient selection is essential for the success of the technique. In daily clinical practice, multidetector computed CT, which allows a three dimensional (3D) reconstruction of the aortic root, its dimensions, and calcium distribution, plays an important role.
